# Prevalence of *Fasciola gigantica* infection in slaughtered animals in south-eastern Lake Chad area in relation to husbandry practices and seasonal water levels

**DOI:** 10.1186/1746-6148-10-81

**Published:** 2014-04-04

**Authors:** Vreni Jean-Richard, Lisa Crump, Abbani Alhadj Abicho, Ngandolo Bongo Naré, Helena Greter, Jan Hattendorf, Esther Schelling, Jakob Zinsstag

**Affiliations:** 1 Swiss Tropical and Public Health Institute, Basel, Switzerland; 2 University of Basel, Basel, Switzerland; 3 Centre de Support en Santé Internationale, N’Djamena, Chad; 4 Institut de Recherche en Elevage pour le Développement, N’Djamena, Chad

**Keywords:** Fasciolosis, Lake Chad, Mobile pastoralists, Slaughter slabs

## Abstract

**Background:**

Fasciolosis has been described in sub-Saharan Africa in many accounts, but the latest reports from Chad are from the 1970s. Mobile pastoralists perceive liver parasites as a significant problem and think that proximity to Lake Chad can lead to infection. This study aimed to assess the importance of liver fluke infections in mobile pastoralists’ livestock in the south-eastern Lake Chad region.

In 2011, all animals presented at three slaughter slabs near Gredaya in the south-eastern Lake Chad area were examined for infection with *Fasciola spp.* during routine meat inspections.

**Results:**

This study included 616 goats, 132 sheep and 130 cattle. The prevalence of adult *Fasciola gigantica* was 68% (CI 60-76%) in cattle, 12% (CI 10-16%) in goats and 23% (CI 16-30%) in sheep. From all infected animals (n = 200), 53% (n = 106) were classified as lightly infected with 1-10 parasites, 18% (n =36) as moderately infected with 11-100 parasites and 29% (n = 58) as heavily infected with more than 100 parasites per animal.

Animals grazing close to the shores of Lake Chad had a much higher risk of infection (prevalence =38%; n = 329) than animals not feeding at the lake (n = 353), with only one goat being positive (prevalence = 0.28%).

The ethnic group of the owner was a strong determinant for the risk of infection. Ethnic group likely served as a proxy for husbandry practices. Geospatial distribution showed that animals originating from areas close to the lake were more likely to be infected with *F. gigantica* than those from more distant areas.

**Conclusions:**

Livestock belonging to ethnic groups which traditionally stay near surface water, and which were reported to feed near Lake Chad, have a high risk of infection with *F. gigantica.* Pastoralist perception of fasciolosis as a priority health problem was confirmed.

Regular preventive and post-exposure treatment is recommended for animals grazing near the lake. However, further economic analysis is needed.

## Background

Fasciolosis is a parasitic disease of herbivorous mammals caused by trematodes of the genus *Fasciola*. In livestock, it causes severe reductions in milk and meat yield as well as losses due to decreased fertility [1,2]. The host animals become infected with *Fasciola* metacercariae when they ingest contaminated vegetation close to or within water bodies. Swamp areas and seasonally flooded areas at the borders of Lake Chad provide an optimal habitat for the parasites and the intermediate hosts, which are freshwater snails of the family Lymnaeidae.

In sub-Saharan Africa, infections with *Fasciola gigantica* have often been described [2-5]. In the Lake Chad area, two previous studies from Niger and Cameroon [6,7] have described the disease, but there is no publication from the Chadian side of the lake. *Fasciola gigantica* has been reported in Chadian cattle and small ruminants in a Central African study of wild ruminants [8] and in a treatment study on Chadian cattle [9]. *Fasciola hepatica* infection has not been reported in Chad.

Fasciolosis is perceived as a significant animal health problem by the mobile pastoralist population in the south-eastern Lake Chad area, particularly since other diseases like bovine pleuropneumonia, against which vaccination is compulsory, are better controlled. Most pastoralist camp leaders expressed concerns about their animals grazing on contaminated pastures in close proximity to water bodies of Lake Chad during a participatory research needs assessment [10]. Some pastoralists are aware that it is possible to treat animals for liver flukes with anti-parasitic drugs, but access to quality drugs is difficult in the remote zones [11].

The majority of the income for mobile pastoralists in this area is generated by selling milk and animals at local markets [12], so the adverse economic impact of fasciolosis is of primary importance. The mobile pastoralists observe the parasites when they slaughter animals and are aware that this is a cause of reduced milk production and body weight. This study was initiated to investigate the mobile pastoralists’ priority concern of fasciolosis in their livestock.

## Methods

### Study zone and population

The south-eastern Lake Chad area is densely populated by sedentary people as well as by mobile communities of different ethnic groups during the dry season, from October to June. In this paper, we describe the ethnic groups using the names as given by the local communities. Kanembou are mainly sedentary, while Arabs are semi-nomadic, moving towards the lake at the end of the dry season when pasture becomes scarce around their villages. Peul and Gorane communities may be mobile or sedentary, although most large-scale cattle owners are mobile, including the entire family and all of their livestock. Peul is synonymous with the term Foulbe and Fulani (English). Peul herders graze their animals in close proximity to the lake shore, with the animals often feeding on grass in shallow water. The Kouri pastoralists utilise pasture areas that partially overlap with the Peul, primarily herding their cattle on accessible islands within Lake Chad. In contrast, Gorane do not stay close to the lake, instead capitalising on highly developed well building skills for access to water.

### Sampling strategy

Each week from January to December 2011, the livers of all slaughtered animals were examined for the presence of *Fasciola spp* during routine meat inspections at three slaughter slabs (Gredaya, Sidje and Bache Djani) in the administrative district of Gredaya at the south-eastern border of Lake Chad. The parasite burden was established by incising the liver along the bile ducts, according to the usual local meat inspection process. No further pathological assessment was made as the study took place during routine meat inspection by the local veterinary delegate. A semi-quantitative estimation of the number of flukes was made based on the number of parasites counted in the exposed surfaces. Infection was classified as being light (1-10 flukes), moderate (11-100) or heavy (>100) in intensity. Most of the off-take from the local herds was animals which were slaughtered and sold at the weekly markets in Gredaya, Sidje and Bache Djani, and all of these were included in the study. Animals slaughtered in households were not examined. The number of examined animals varied from 1-22 per day of observation. The veterinarian interviewed the owner of each animal presented for slaughter, completing a short questionnaire. Information included the origin of the animal, ethnic group of the current owner, animal breed, history of the animal grazing in or in close proximity to Lake Chad and the semi-quantitative level of flukes counted in the liver.

**Figure 1:**
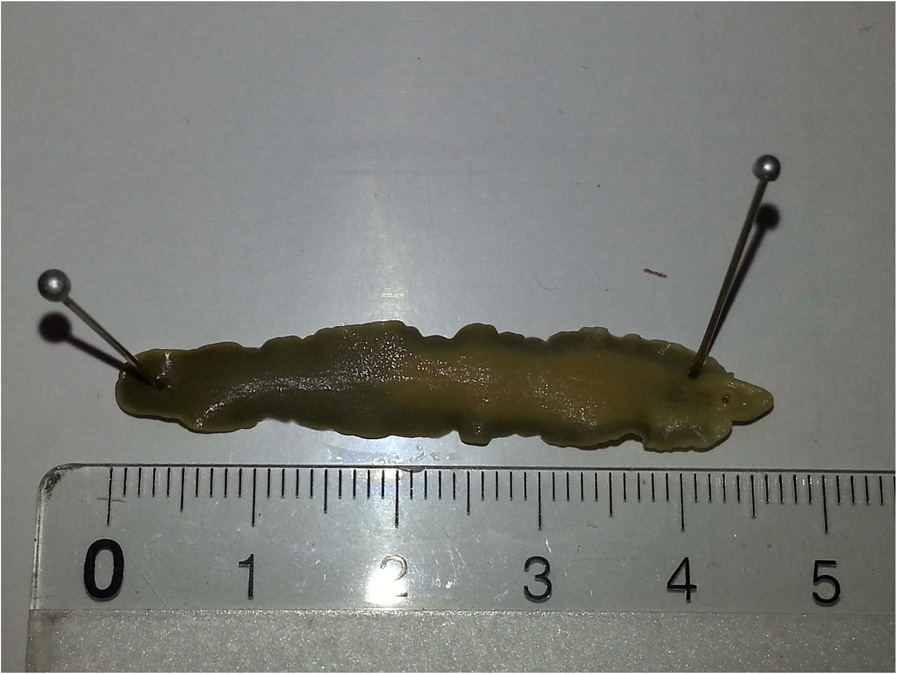
***Fasciola gigantica *** **from a cow in the south-eastern Lake Chad area.**

Morphological analysis and measurement of a subsample of the individual fluke specimens collected from infected animals (Figure 1) was performed at the Laboratoire de Recherches Vétérinaires et Zootechniques in Chad, based on descriptive criteria [13-15], including body size and shape, form of the apical cone and position of the suction cups and ovaries.

The data were double entered in Microsoft® Access 2002 (Microsoft Corp.; Redmond, WA,USA), and compared using Epi Info™ 3.5.1 Data Compare program (Centers for Disease Control and Prevention, Atlanta, GA, USA). Statistical analysis using descriptive statistics and logistic regression was conducted with Stata IC 10.1 (StataCorp LP, College Station, USA). ArcGIS 9.3 (ESRI Inc. ArcMap™ 9.3, Redlands, CA, USA) and Google Earth (Google Inc., Mountain View, CA, USA) were used for mapping and spatial analysis.

## Results

A total of 880 animals were examined. Two animals were excluded because questionnaires were not completed. Data from the remaining 130 cows, 616 goats and 132 sheep were analysed. The distribution of livestock species by owner ethnic group is shown in Table 1.

**Table 1:** Sample size of species and ethnic groups of the owners

	**n**	**%**	**Arab**	**%**	**Peul**	**%**	**Gorane**	**%**	**Kanembou**	**%**	**Kouri**	**%**
Cattle	130	15%	10	8%	85	65%	28	22%	1	1%	6	5%
Goats	616	70%	278	45%	133	22%	133	22%	72	12%	0	0%
Sheep	132	15%	27	20%	56	42%	47	36%	2	2%	0	0%
Total	878	100%	317	36%	274	31%	208	24%	75	9%	6	1%

*Fasciola gigantica* specimens (n = 11) measured between 2 and 5 cm, with a mean size of 3.2 cm.

The prevalence of *F. gigantica* was 68% (95% CI 60-76%) in cattle, 12% (95% CI 10-16%) for goats and 23% (95% CI 16-30%) for sheep. The analysis revealed a strong relationship (p < 0.001) between grazing at the lake and *F. gigantica* infection. Not feeding at the lake was a protective factor, and only one animal reported as not grazing near the lake was infected with *F. gigantica* (0.28%) (Table 2).

**Table 2:** **Prevalence for infections with *F. gigantica *** **by species and stratified for grazing area**

	**Grazing LC**	**pos**	**%**	**neg**	**%**	**Total**	**Prev**	**p**	**OR**	**CI (95%)**
All animals	yes	198	38%	329	62%	527	22.6%	<0.001	212	29.5-1520
	no	1	0%	352	100%	353		Baseline		
Cattle	yes	89	93%	7	7%	96	68.5%	n.a. perfect prediction		
	no	0	0%	34	100%	34				
Goats	yes	79	22%	277	78%	356	13.0%	<0.001	74	10.2-534.6
	no	1	0%	259	100%	260		Baseline		
Sheep	yes	30	41%	43	59%	73	22.7%	n.a. perfect prediction		
	no	0	0%	59	100%	59				

The highest prevalence was seen in cattle from the Kouri ethnic group (100%, n = 6), and livestock owned by Peul also showed high prevalence (95% for cattle, 33% for goats, 48% for sheep). None of the Gorane cattle were reported to have grazed in the lake, and none were positive for *F. gigantica*. Of all Gorane animals (n = 208), only one goat was infected with *F. gigantica*. There was no infection with *F. gigantica* in animals from Kanembou breeders (n = 75). The prevalence in Arab livestock (13%, n = 317) ranged in between those grazing near lake water and those not near the lake (Table 3).

**Table 3:** **Prevalence of *F. gigantica *** **in different livestock species by ethnic group of the owner**

**Ethnic group**		**pos**	**%**	**neg**	**%**	**Total**	**p**	**OR**	**CI (95%)**
Gorane		1	0%	207	100%	208	0.001	0.03	0-0.2
Peul		152	55%	122	45%	274	<0.001	8.6	5.7-12.9
Arab		40	13%	277	87%	317	Baseline		
Kanembou		0	0%	75	100%	75	n.a. perfect prediction		
Kouri		6	100%	0	0%	6	n.a. perfect prediction		
Cattle	Gorane	0	0%	28	100%	28	n.a. perfect prediction		
	Peul	81	95%	4	5%	85	<0.001	81	12.8-513.2
	Arab	2	20%	8	80%	10	Baseline		
	Kanembou	0	0%	1	100%	1	n.a. perfect prediction		
	Kouri	6	100%	0	0%	6	n.a. perfect prediction		
Goats	Gorane	1	1%	132	99%	133	0.04	0.05	0-0.4
	Peulh	44	33%	89	67%	133	<0.001	3.4	2.1-5.7
	Arab	35	13%	243	87%	278	Baseline		
	Kanembou	0	0%	72	100%	72	n.a. perfect prediction		
Sheep	Gorane	0	0%	47	100%	47	n.a. perfect prediction		
	Peul	27	48%	29	52%	56	0.003	7.4	2.0-27.6
	Arab	3	11%	24	89%	27	Baseline		
	Kanembou	0	0%	2	100%	2	n.a. perfect prediction		

Among all positive animals (n = 200), 53% (n = 106) were classified as lightly infected (1-10 parasites), 18% (n =36) as moderately infected (11-100 parasites) and 29% (n = 58) as heavily infected (> 100 parasites). In cattle, 19% of infections (n = 17) were light, 20% (n = 18) moderate and 61% (n = 54) heavy; in goats, 80% (n = 65) were light, 17% (n = 14) moderate and 2% (n = 2) heavy; and in sheep, 80% (n = 24) light, 13% (n = 4) moderate and 7% (n = 2) heavy. There was a significant difference between the degree of infection in cattle and small ruminants. Sheep and goats had very similar prevalence and burdens.

The prevalence in cattle in Sidje, the slaughter slab closest to Lake Chad, was significantly higher than in Gredaya (p = 0.003). Seasonal trends indicate a lower prevalence between the months of August to October, comprising the rainy season, compared to the rest of the year in all species.

### Geospatial distribution

**Figure 2:**
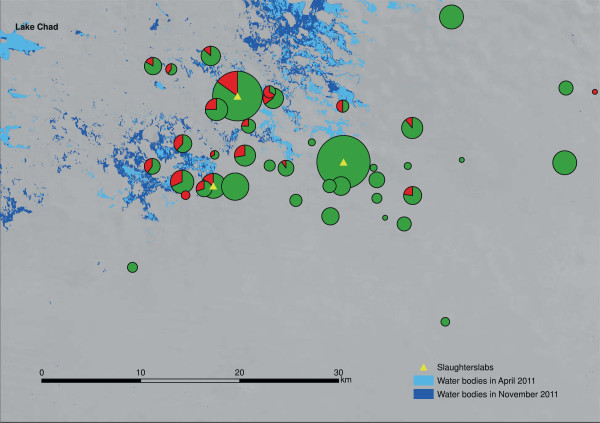
**Prevalence of *F. gigantica *in slaughtered goats by village of origin.** Legend: prevalence rate according to village of origin coordinates, circle size corresponds to the number of animals, red indicates proportion positive for *Fasciola gigantica*.

The prevalence rates were plotted according to the coordinates of the villages of origin to show the geospatial distribution of animals and the proportion of positive animals. The size of the circle corresponds to the number of animals originating in each village (Figure 2 shows the data for goats, data for cattle are shown in Additional file 1: Figure S1, data for sheep are shown in Additional file 2: Figure S2). There is a notable relationship between proximity to the lake and infection with *F. gigantica* in all three species.

## Discussion

This is the first publication on *Fasciola* infection in cattle, sheep and goats in the Lake Chad area of Chad. The results support a relationship between the infection of livestock with *F. gigantica* and the ethnic group of the livestock holder. The ethnic group likely serves as a proxy for the type of animal husbandry practiced [10]. The Kouri cattle, which were kept on islands in the lake, were 100% positive for *F. gigantica*. Although the sample size was very small (n = 6), nonetheless there was clearly a high prevalence in these animals. The livestock kept by Peul, who utilise pastures close to the lake and its seasonal extensions, also showed a high prevalence (55% overall, 95% for cattle). In contrast was the low prevalence found in the Gorane and Kanembou livestock. The Gorane pastoralists do not move close to the lake, but stay in drier areas to the east. The Kanembou culture and husbandry practices are, in general, similar to the Gorane, although in the study zone, the majority of Kanembou were sedentary rather than mobile. The prevalence found in Arab livestock ranged in between that found in the other ethnic groups (Kouri/Peul and Gorane/Kanembou). This finding is supported considering that Arab cattle breeders in the zone were semi-nomadic, only moving their animals towards the lake at the end of the dry season, when the pastures around their villages were depleted.

There was a notable relationship between proximity to the lake and infection with *F. gigantica* in all three species. The geospatial distribution and the analysis of grazing patterns strongly suggest that Lake Chad is the source of infection. This would also explain the observed seasonal trend, which is likely due to migration away from the lake during the rainy season when grass is more widely available, potentially reducing exposure to the contaminated areas close to the lake. Further research is currently continuing to establish the seasonal dynamics of *F. gigantica*. The results of this study support a strong recommendation, for Kouri and Peul livestock, for treatment against *F. gigantica* infection with an initial prophylactic dose when entering the lake region and a second dose at the beginning of the rainy season, or when leaving the area. This type of programme could reduce pasture contamination and the effects on livestock productivity, particularly for Arab livestock that is not continuously grazed near the lake. In contrast, no preventive treatment is necessary for Gorane and Kanembou livestock that are grazed in areas not near the lake. Because they are not in proximity to open water, these animals have a negligible risk of infection. Our recommendation is in line with that of the local veterinarian in the Gredaya administrative district, who recommended treatment every three months as long as animals were kept near the lake.

It was noted that prevalence and degree of infection differ between species. This is likely due to feeding patterns as well as specific husbandry practices. Small ruminants avoid wet areas, instead preferring to graze and browse on dry ground. However, grazing dry pastures is not completely protective, as *Fasciola* metacercariae can remain viable for some time on vegetation and in some of the intermediate host snails of the genus *Lymnaea* in previously flooded areas [13,14]. Pastoralists in the study area reported that they kept their small ruminants away from the more humid areas near the water as long as possible to decrease the risk of infection, as also noted by Tager-Kagan in the 1970s [6].

In this study, sheep and goats had comparable infection intensities. The similar burden in sheep and goats could indicate similar susceptibility to infection and/or result from use of comparable feeding areas. Although sheep commonly graze ground cover, while goats typically browse shrubs and trees, the Lake Chad region is now densely populated with herds and subject to increased agricultural cultivation, so there are relatively few shrubs, particularly at the end of the dry season.

The higher prevalence noted in cattle at the slaughter slab in Sidje is likely because many Peul pastoralists pass by this village when leaving the lake or stay nearby during the rainy season.

In this study, meat inspection was performed according to the routine local inspection procedure, which consisted of one long transverse cut in the liver along the bile ducts. The method of examination was a limitation to this study in that it provided only a semi-quantitative measurement of the parasite burden. While it would have been ideal to examine the entire organ by cutting it into small pieces to visualise all biliary ducts, the cost to purchase every liver precluded such a method. Using the standard meat inspection approach, it is possible that some animals with few parasites might have been misclassified as not infected. Also, particularly in cattle due to the large liver size, multiple incisions might have shown a higher number of parasites. Therefore, the prevalence and degree of *F. gigantica* infection intensity might have been underestimated using the standard, locally available, semi-quantitative evaluation method employed in this study, but the exposure patterns revealed are nonetheless significant and valid, despite a potentially decreased sensitivity of this method.

Although fasciolosis is increasingly being recognised as a human public health issue [15,16], there is very little literature on *F. gigantica* from Chad or the Lake Chad region, with the most recent dating from the late 1970s [6-8]. At that time, the recommended control measure was routine deworming treatment of livestock once or preferably twice per year, before and after the rainy season [6,9]. Based on the results of the present study, this recommendation should still be implemented for herds grazing near Lake Chad. Further cost-benefit analysis is warranted, as findings would provide evidence for information campaigns and policy development. It is also recommended to assess pastoralist’s access to and the quality of available treatments for fasciolosis in remote areas.

## Conclusions

This research quantifies the prevalence of *F. gigantica* in slaughtered livestock in south-eastern Lake Chad area and provides a semi-quantitative assessment of the burden of infection.

The results showed that animals which had grazed in close proximity to the lake and its seasonal extensions had a high risk of infection. Cattle of Peul and Kouri ethnic groups were most affected. These groups keep their animals at the shore of Lake Chad or on islands within the lake.

The study confirms the pastoralists’ perceptions of disease priorities with fasciolosis as an important health problem.

Treatment against fasciolosis is recommended for animals grazing near or at the lake, and further economic analysis of such treatment is warranted.

The data set supporting the results of this article is available in Additional file 3.

## Competing interests

The authors declare that they have no competing interests.

## Authors’ contributions

VJR supervised data collection in situ, entered and analysed the data and drafted the manuscript. LC contributed to the realisation of the study and drafted the manuscript. AAA assisted with data entry and coordinated the collection and transport of data. NBN and HG participated in the conceptualisation and realisation of the study and contributed expert opinions to the draft. JH provided statistical knowledge and contributed to statistical analysis. ES added expert knowledge from earlier research in the area. JZ engaged in the conceptualisation of the study and gave general supervision throughout the study. All authors read and approved the final manuscript.

## Supplementary Material

Additional file 1: Figure S1 Prevalence of *F. gigantica* in slaughtered cattle by village of origin. Legend: prevalence rate according to village of origin coordinates, circle size corresponds to the number of animals, red indicates proportion positive for *Fasciola gigantica*. Click here for file

Additional file 2: Figure S2 Prevalence of *F. gigantica* in slaughtered sheep by village of origin. Legend: prevalence rate according to village of origin coordinates, circle size corresponds to the number of animals, red indicates proportion positive for *Fasciola gigantica*. Click here for file

Additional file 3 **Original dataset from questionnaires, -9 always means missing data.** Description of columns: ID: ID of record. Date: Date of slaughtering. Abbatoir: ID of slaughterslab 1 = Gredaya, 2 = Sidje, 3 = Bache Djani. Species: Livestock species 1 = cattle 2 = goats 3 = sheep 8 = camels. Origin: Village or well of origin. CoordinatesN: Latitude of the coordinates of the specific village. CoordinatesE: Longitude of the coordinates of the specific village. PastureLake: 1 = the animal has been feeding in close proximity to the lake, 2 = the animal has not been feeding at the lake. Presence_Flukes: 1 = F. gigantica was found in this animal, 2 = F.gigantica was not found in this animal. Infection_Intensity: 0 = no infection; 1 = light infection (1-10 parasites); 2 = medium infection (11-100 parasites); 3 = above 100 parasites (heavy infection). Group_ethn: Ethnic group of livestock owner: 1 = Arab, 2 = Peul; 3 = Gorane; 4 = Kanembou; 5 = Kouri. Click here for file
