# Supplementary figures and images for: Prevalence of Fasciola gigantica infection in slaughtered animals in south-eastern Lake Chad area in relation to husbandry practices and seasonal water levels
Source: BMC Vet Res. 2014 Apr 4;10:81. doi: 10.1186/1746-6148-10-81 (PMC4022263; doi:10.1186/1746-6148-10-81)

Lake Chad

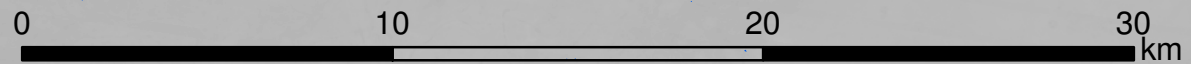

- 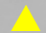 Slaughterslabs
- 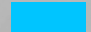 Water bodies in April 2011
- 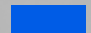 Water bodies in November 2011

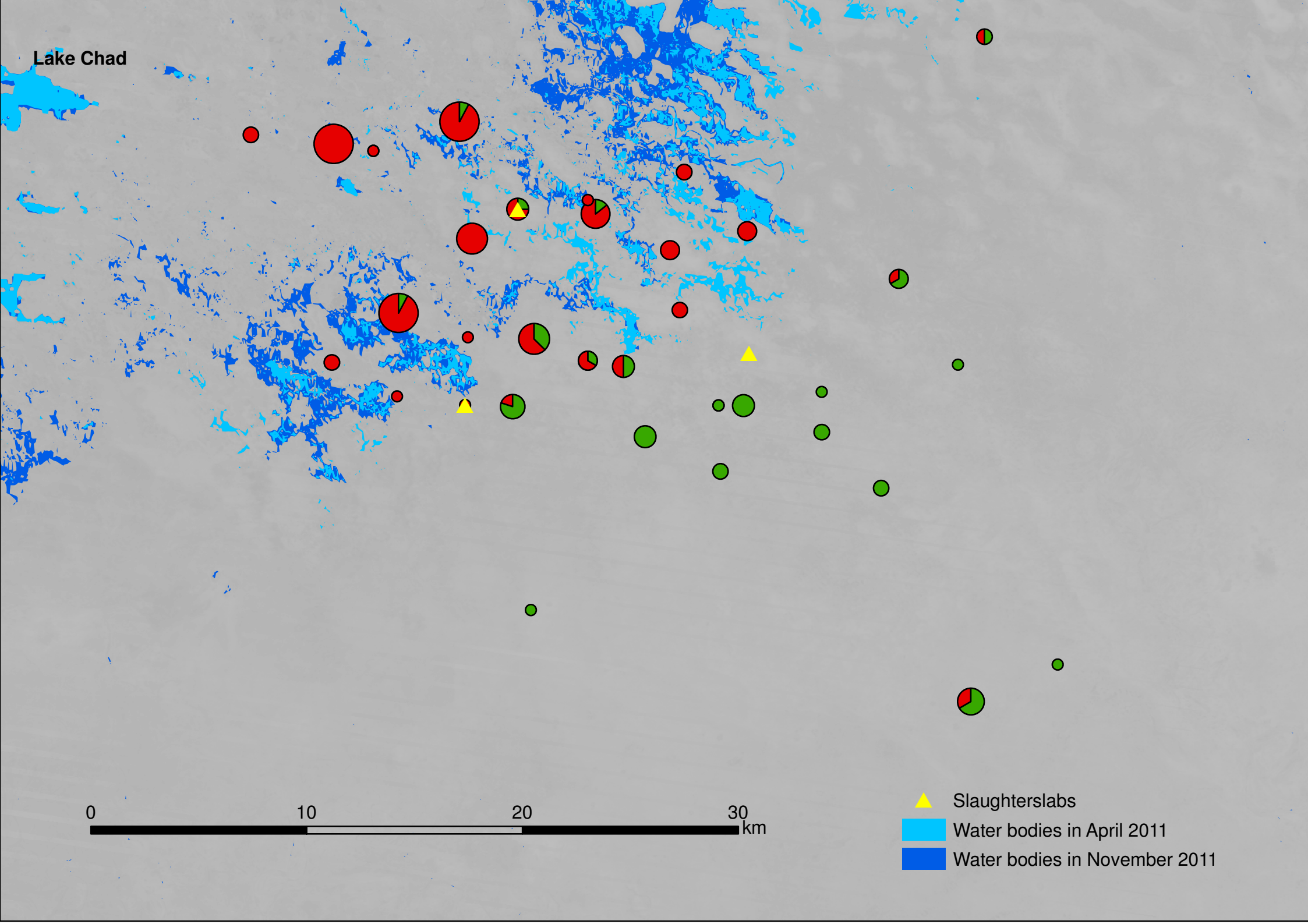

Supplement: Additional file 1: Figure S1 — Prevalence of F. gigantica in slaughtered cattle by village of origin. Legend: prevalence rate according to village of origin coordinates, circle size corresponds to the number of animals, red indicates proportion positive for Fasciola gigantica. [file 1746-6148-10-81-S1.pdf]

Lake Chad

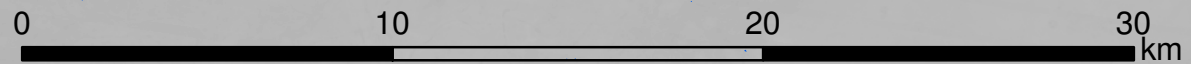

- 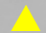 Slaughterslabs
- 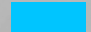 Water bodies in April 2011
- 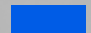 Water bodies in November 2011

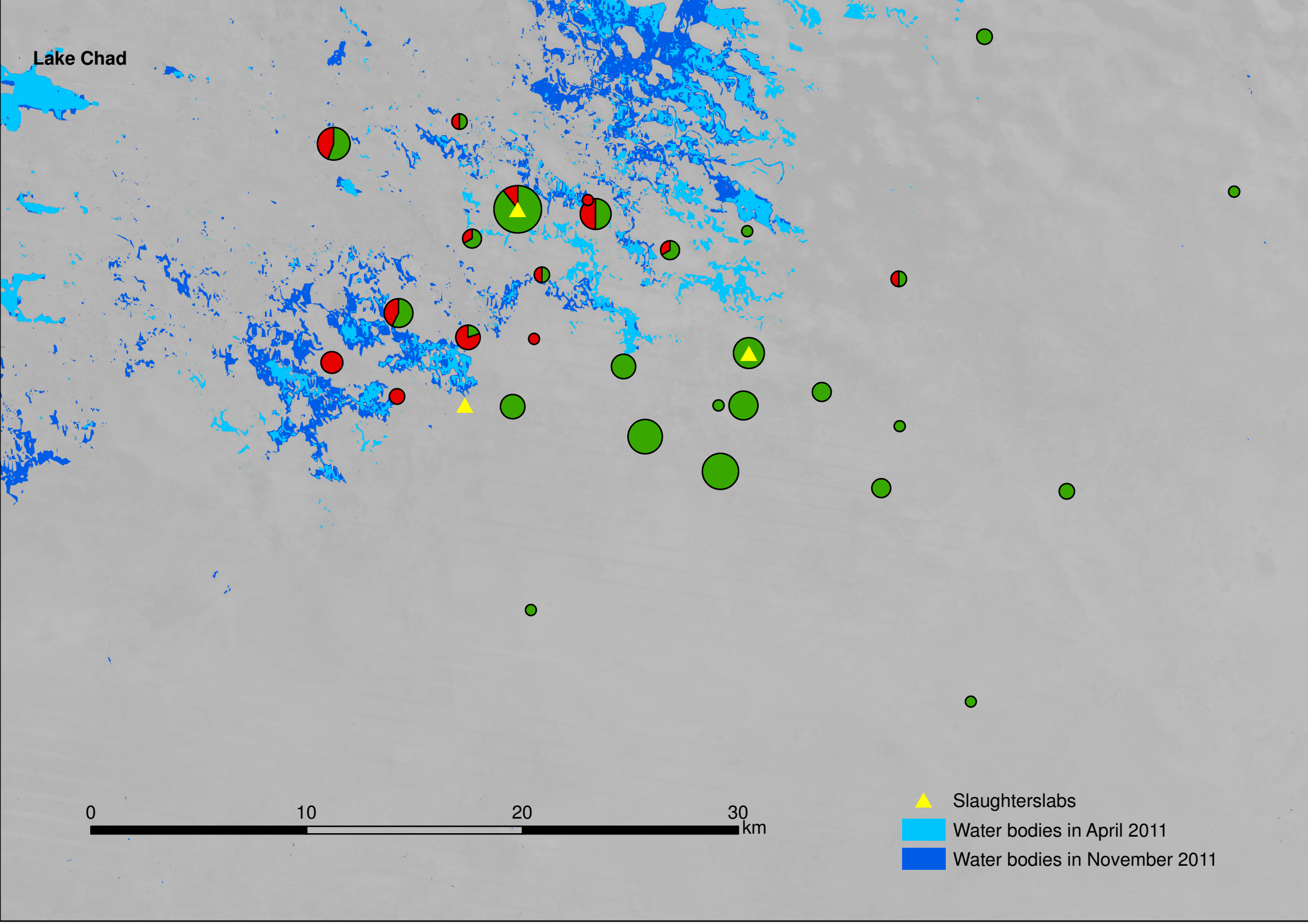

Supplement: Additional file 2: Figure S2 — Prevalence of F. gigantica in slaughtered sheep by village of origin. Legend: prevalence rate according to village of origin coordinates, circle size corresponds to the number of animals, red indicates proportion positive for Fasciola gigantica. [file 1746-6148-10-81-S2.pdf]
